# Gimme shelter: how *Vibrio fischeri* successfully navigates an animal’s multiple environments

**DOI:** 10.3389/fmicb.2013.00356

**Published:** 2013-11-29

**Authors:** Allison N. Norsworthy, Karen L. Visick

**Affiliations:** Department of Microbiology and Immunology, Loyola University Medical CenterMaywood, IL, USA

**Keywords:** *Vibrio fischeri*, *Euprymna scolopes*, symbiosis, biofilm, chemotaxis, antimicrobials, bioluminescence

## Abstract

Bacteria successfully colonize distinct niches because they can sense and appropriately respond to a variety of environmental signals. Of particular interest is how a bacterium negotiates the multiple, complex environments posed during successful infection of an animal host. One tractable model system to study how a bacterium manages a host’s multiple environments is the symbiotic relationship between the marine bacterium, *Vibrio fischeri,* and its squid host, *Euprymna scolopes*. *V. fischeri* encounters many different host surroundings ranging from initial contact with the squid to ultimate colonization of a specialized organ known as the light organ. For example, upon recognition of the squid, *V. fischeri* forms a biofilm aggregate outside the light organ that is required for efficient colonization. The bacteria then disperse from this biofilm to enter the organ, where they are exposed to nitric oxide, a molecule that can act as both a signal and an antimicrobial. After successfully managing this potentially hostile environment, *V. fischeri* cells finally establish their niche in the deep crypts of the light organ where the bacteria bioluminesce in a pheromone-dependent fashion, a phenotype that *E. scolopes* utilizes for anti-predation purposes. The mechanism by which *V. fischeri* manages these environments to outcompete all other bacterial species for colonization of *E. scolopes* is an important and intriguing question that will permit valuable insights into how a bacterium successfully associates with a host. This review focuses on specific molecular pathways that allow *V. fischeri* to establish this exquisite bacteria–host interaction.

## INTRODUCTION

Bacteria are remarkably successful organisms because they can effectively sense and acclimatize to a wide variety of environments. This domain of life can flourish in habitats ranging from deep sea hydrothermal vents, to scum growing on a lakebed, and to the gastrointestinal tracts of humans (Orcutt et al., 2011; Salzman, 2011). To thrive in particular environments, bacteria use molecular signaling cascades that recognize extracellular signals and activate intracellular pathways, often leading to a change in gene expression. These changes in gene regulation allow a cell to manage the array of extracellular signals and adapt accordingly.

Of particular interest are the signaling cascades that permit a microbe to cope with the multiple environments found within a eukaryotic host. These pathways presumably sense changing environmental factors such as osmolarity, fluctuating nutrient sources, other microorganisms, antimicrobials, and components of the immune system. Furthermore, a bacterium must integrate the multiple inputs to identify which location, if any, is an appropriate niche. To ask in-depth questions about signaling pathways involved in host colonization, researchers often study “simplified” model systems, in which only one or a few bacterial species successfully infect a host (McFall-Ngai et al., 2013). One model system used for this purpose is the symbiosis between the luminescent marine bacterium, *Vibrio fischeri*, and its nocturnal squid host, *Euprymna scolopes*. In this symbiosis, *V. fischeri* is the only bacterium capable of colonizing a specialized symbiotic organ, the light organ. This monospecific association permits researchers to ask deeply reductionist questions about bacteria/host interactions, and has provided insights into how a single bacterial species controls its gene expression to cope with different host environments.

**FIGURE 1:**
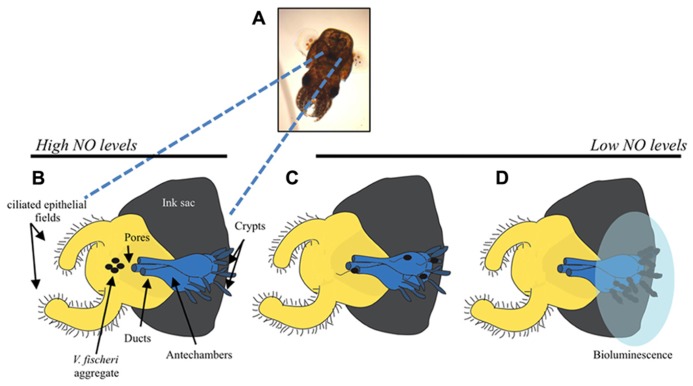
**Steps of *E. scolopes* colonization by the luminescent bacterium, *V. fischeri*.** **(A)** Image of a juvenile *E. scolopes.* The bi-lobed light organ can be seen as a black structure in the mantle cavity. **(B)** Cartoon depicting one lobe of the light organ with the ink sac (gray), ciliated epithelial cells (yellow), and internal regions of the light organ (blue). Before the initial contact with *V. fischeri* (black ovals), *E. scolopes* produces the reactive nitrogen radical, nitric oxide (NO), which it subsequently down-regulates after exposure to the bacteria. Initiation of colonization requires that *V. fischeri* cells form a biofilm-like aggregate around the pores to the light organ. Motility is not required for biofilm formation. **(C)** After aggregation, *V. fischeri* cells utilize flagella to migrate into the pores, through the ducts and antechamber, and to establish their niche in the crypt spaces. **(D)** Once in the crypts, *V. fischeri* lose their flagella and grow to a sufficient cellular density that allows for the induction of bioluminescence genes (transparent blue oval represents luminescence). Figure modified from Nyholm and McFall-Ngai (2004).

There are a number of experimentally tractable steps involved in colonization of *E. scolopes*, many of which are facilitated by known signaling pathways in *V. fischeri*. Newly hatched squid are aposymbiotic and must acquire *V. fischeri* cells from the surrounding seawater (Wei and Young, 1989). Ventilation by the squid brings seawater and any bacterial cells into the mantle cavity where the light organ is located (**Figure 1**). To aid in the recruitment of bacteria, the surface of the light organ has epithelial fields with cilia that circulate the seawater (McFall-Ngai and Ruby, 1991). This motion draws cells toward six pores leading into the light organ. In as little as 1 h, *V. fischeri* and other Gram-negative bacteria make contact with cilia and then form biofilm-like aggregates around the cilia and within mucous shed by the host in response to bacterial peptidoglycan (Nyholm et al., 2000; Altura et al., 2013). During these early processes, *V. fischeri* cells secrete molecules, known as microbe-associated molecular patterns (MAMPs), that induce morphological changes and alterations in gene expression in the squid, thereby resulting in a host environment actively shaped by the symbiont (for reviews, see Nyholm and McFall-Ngai, 2004; Visick and Ruby, 2006; McFall-Ngai et al., 2012) Ultimately, *V. fischeri* cells dominate over other bacteria within the aggregate through unknown mechanisms (Nyholm and McFall-Ngai, 2003; Altura et al., 2013). After these initial interactions, *V. fischeri* cells then leave the aggregate, enter into the ducts of the light organ, travel through antechambers (spaces not permissive for colonization), and arrive within the crypts, the sites of colonization. Within the location of these different host tissues, *V. fischeri* cells are subjected to host-derived stresses such as reactive oxygen species (ROS) and reactive nitrogen species (RNS), that they must sense and resist (Tomarev et al., 1993; Weis et al., 1996; Small and McFall-Ngai, 1999; Davidson et al., 2004). When the bacteria finally reach the crypt spaces, they grow to high cell density and begin to bioluminesce. Bioluminescence is a key component of the symbiosis: in exchange for a nutrient-rich niche, the bacteria provide light that the squid can use to avoid predation (Ruby, 1996; Jones and Nishiguchi, 2004). Every day at dawn, the squid expel ~95% of the *V. fischeri* cells back into the seawater environment, leaving the remaining *V. fischeri* cells to repopulate the light organ (Lee and Ruby, 1994). It has been suggested that this process allows the squid to prevent bacterial overgrowth, thus relieving the burden of carrying a dense growth of bacterial cells (Ruby and Asato, 1993).

Research in the *V. fischeri/E. scolopes* symbiosis field has identified a number of molecular signaling pathways that facilitate the various steps of colonization. A few of these pathways within *V. fischeri* include controlling biofilm formation during the aggregation step, motility and chemotaxis to propel and direct the bacteria toward the crypts, ROS and RNS management during all steps of colonization, and bioluminescence within the light organ. This review will focus on these well-known signaling cascades, although it should be noted that other important pathways exist within *V. fischeri* to promote the symbiosis (reviewed in Nyholm and McFall-Ngai, 2004; Visick and Ruby, 2006; Dunn, 2012; McFall-Ngai et al., 2012; Stabb and Visick, 2013).

## INITIATING THE SYMBIOSIS: BIOFILM FORMATION

The first step of colonization requires that *V. fischeri* cells come into the vicinity of and sense the presence of the squid. This seemingly simple task, however, can be considered a limiting factor in colonization. For example, within the Hawaiian water where *E. scolopes* reside, *V. fischeri* constitute ~100 to 1500 cells per ml of seawater representing as little as 0.01% of the total bacterial population (Lee and Ruby, 1994; Nyholm and McFall-Ngai, 2004). Furthermore, the light organ is not openly exposed to the seawater; instead *E. scolopes* vents seawater through its mantle cavity and across the entrance to the light organ. It has been estimated that a miniscule volume of seawater (1.3 μl) and thus only a few *V. fischeri* cells enter the mantle during each half-second ventilation (Nyholm et al., 2000). Additionally, one *V. fischeri* cell constitutes only one-millionth the volume of the mantle cavity (McFall-Ngai and Montgomery, 1990; Nyholm et al., 2000). Theoretically, *V. fischeri* cells would have to locate all six pores in a brief amount of time before they are expelled from this cavity (Nyholm et al., 2000; Nyholm and McFall-Ngai, 2004). So how does this microbe manage the transition from seawater to squid?

*V. fischeri* cells do not immediately enter the light organ during ventilation; they first interact with mucous and cilia on the host’s epithelial cells, and then they begin to coalesce into a bacterial aggregate (Nyholm et al., 2000; Altura et al., 2013; **Figure 1**). *V. fischeri* strains that fail to form this aggregate or form an enhanced aggregate either fail to colonize the squid or exhibit an enhancement of colonization, respectively (Nyholm et al., 2000; Millikan and Ruby, 2002; Yip et al., 2006; Morris and Visick, 2013). Because this stage of colonization is a critical step in establishing the symbiosis, much research has focused on the mechanisms by which *V. fischeri* cells form these squid-specific aggregates. Of note is the discovery of the 18 gene *syp* (*sy*mbiosis *p*olysaccharide) locus, which was found to be important for the formation of a biofilm, or a community of cells encased in a protective matrix often composed of polysaccharides and other macromolecules (Yip et al., 2005, 2006). The *syp* locus encodes proteins predicted to regulate, produce, or transport the biofilm polysaccharide, and most of the *syp* genes are critical for both *in vitro* biofilm formation colonization (Yip et al., 2005; Shibata et al., 2012). Perhaps not surprisingly, given its importance for initiating the symbiosis, there are layers of controls in place that regulate the formation of this biofilm (Yip et al., 2006; Morris and Visick, 2013).

**FIGURE 2:**
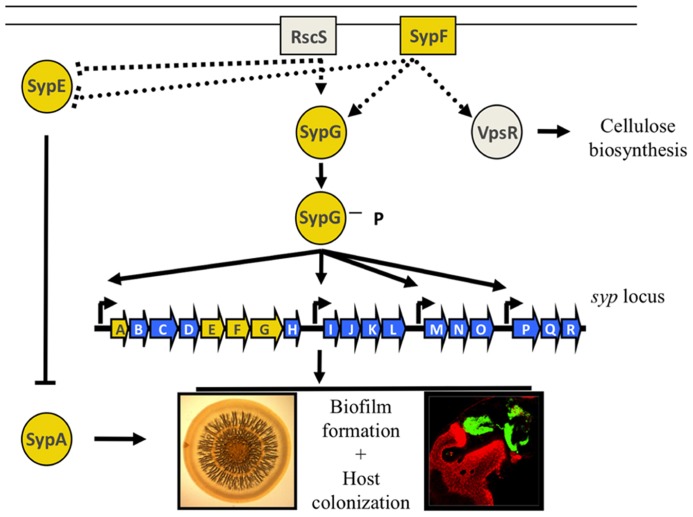
**Regulation of biofilm formation in *V. fischeri*.** Two-component regulators control the production of the Syp biofilm. RscS and SypF are proposed to function as sensor kinases, resulting in the phosphorylation of the two downstream response regulators, SypE and SypG. SypG functions as a transcription factor to control expression of the *syp* locus at its four promoters, while SypE functions downstream of *syp* transcription to control the phosphorylation state of the small STAS domain protein, SypA. SypF is also predicted to control the activity of a RR VpsR putatively involved in cellulose biosynthesis. Biofilms can be assessed *in vitro* as a wrinkled colony on an agar plate, or *in vivo* as a bacterial aggregate that forms on the surface of the light organ. Adapted from (Visick, 2009).

Production of the Syp biofilm is controlled by a two-component signaling (TCS) cascade, a ubiquitous class of signaling pathways consisting of two types of proteins: a sensor kinase (SK) that receives input signals from the environment, causing it to autophosphorylate, and a response regulator (RR), a protein that receives the phosphoryl group from the SK (reviewed in Stock et al., 2000). This phospho-transfer often changes the activity of the effector domain on the RR, thus leading to a cellular response. The particular TCS pathway that controls production of the Syp biofilm is more complicated than canonical TCS cascades; it contains at least two SKs and two RRs (**Figure 2**; reviewed in Visick, 2009). Overexpression of the SK predicted to be at the top of the hierarchy, RscS, is sufficient to induce biofilm formation *in vitro* and *in vivo* by affecting the activity of two downstream RRs, SypG and SypE (Yip et al., 2006; Hussa et al., 2008; Morris et al., 2011). Phospho-SypG promotes transcription from the four *syp* promoters (Yip et al., 2005; Ray et al., 2013). Unphosphorylated SypE inhibits biofilms at a level below *syp* transcription; however, when SypE is phosphorylated, it functions as a positive regulator (Morris and Visick, 2013). SypE controls biofilm formation by changing the phosphorylation state of a small STAS domain protein, SypA, but the exact function of SypA is unknown (Morris and Visick, 2010, 2013). The *sypA, sypE,* and *sypG* genes are located within the *syp* locus whereas *rscS* is an orphan SK gene hypothesized to be acquired through horizontal gene transfer (Visick and Skoufos, 2001; Mandel et al., 2009). An additional putative SK gene, *sypF*, is located between *sypE* and *sypG*. This location suggests that SypF is yet another SK involved in regulating biofilms. In support of this, an “active” allele of sypF, sypF*, was sufficient to promote biofilms in a *sypG-*dependent manner (Darnell et al., 2008). Surprisingly, SypF*-induced biofilms also required *vpsR,* a putative RR that is predicted to be involved in cellulose biosynthesis (Darnell et al., 2008).

Although much is known about the Syp signaling pathway, there are still outstanding questions that have yet to be fully answered. For example, what are the signals that RscS and SypF recognize? Do these SKs function as separate inputs into downstream regulators? What, if any, is the connection between Syp biofilms and cellulose biosynthesis? Furthermore, the sole function of SypE appears to be controlling the activity of SypA (Morris and Visick, 2013), yet what does SypA do? Lastly, although the ability to form the Syp biofilm is required for aggregate formation, it is not required for outcompeting colonization-incompetent species of bacteria that can also aggregate outside the light organ (Nyholm et al., 2000; Nyholm and McFall-Ngai, 2003; Altura et al., 2013). Hence, what Syp-independent mechanisms establish early specificity in the symbiosis by promoting the dominance of *V. fischeri* cells within this aggregate? The answers to these questions will permit a detailed and mechanistic understanding of a critical, early stage of host colonization.

## TRAVERSING THE TERRAIN OF THE SQUID: MOTILITY AND CHEMOTAXIS

Once *V. fischeri* cells aggregate outside the squid’s light organ, they must leave this matrix-encased biofilm, migrate through the pores against outward water currents produced by beating cilia, and traverse across the antechamber and into the crypt spaces in the organ (McFall-Ngai and Montgomery, 1990; McFall-Ngai and Ruby, 1998; Nyholm and McFall-Ngai, 2004). This migration process requires that *V. fischeri* cells have the capability to move through fluids or across surfaces and to direct this movement toward their final destination. For these processes to occur, *V. fischeri* cells utilize flagella for locomotion and chemotaxis proteins to alter the direction of movement.

### MOTILITY

Flagella are large macromolecular appendages with a membrane-embedded motor. This motor rotates the long flagellar filament using energy from ion gradients across the membrane (Berg, 2003). The number and location of the flagella (polar or peritrichous) vary among bacterial species; *V. fischeri* in particular has a tuft of 1–5 sheathed flagella at one pole (Ruby and Asato, 1993; McCarter, 2001). Studies have demonstrated that flagellar-dependent motility is required for early stages of host colonization; non-motile or hypermotile strains fail to efficiently colonize *E. scolopes* (Graf et al., 1994; Millikan and Ruby, 2002, 2004; Wolfe et al., 2004; Brennan et al., 2013b). Interestingly, although cells begin the colonization process flagellated, they lose these appendages within the light organ, suggesting that motility is not important within this environment (Ruby and Asato, 1993). Before dawn, *V. fischeri* begin to express flagellar genes and, once released from the light organ during venting from the squid at dawn, *V. fischeri* cells again have fully formed flagella (Ruby and Asato, 1993; Wier et al., 2010). These data suggest that *V. fischeri* cells change their flagellation status based on a particular environmental cue. In support of this idea, flagellation and thus motility of *V. fischeri* depends on magnesium, a divalent cation common in seawater; thus, the seawater environment might promote flagellar synthesis (O’Shea et al., 2005). The observation that *V. fischeri* cells are not flagellated within the light organ signifies that this region of the squid might constitute a low Mg^2^^+^ environment; however, the abundance and/or role of Mg^2^^+^ *in vivo* has not been assessed.

**FIGURE 3:**
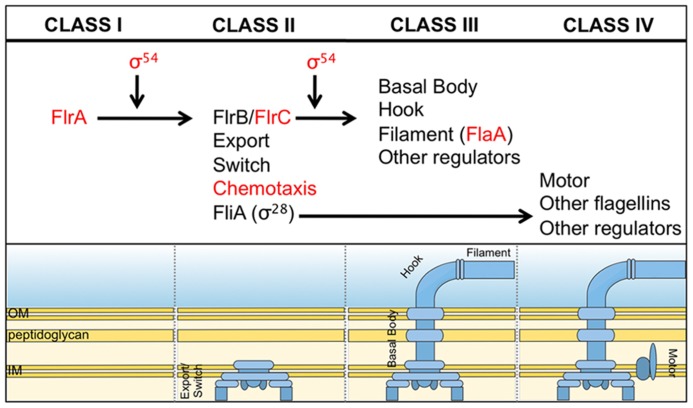
**Predicted flagellar synthesis pathway in *V. fischeri*.** The *V. fischeri* model of flagella gene regulation is based on the pathway elucidated in the related microbe, *V. cholerae* (Prouty et al., 2001). Class I consists solely of the regulator, FlrA, which, together with σ^54^, controls expression of Class II genes. Class II proteins include both those necessary for building the base of the flagellum and also the regulators FlrB, FlrC and σ^28^(FliA). FlrB and FlrC control transcription of Class III genes necessary for synthesis of the distal basal body, hook, and filament, while σ^28^ regulates transcription of Class IV genes involved in the production of motor proteins and other miscellaneous factors. Regulators in red indicate they are important for *V. fischeri* to colonize the squid (Millikan and Ruby, 2003, 2004; Hussa et al., 2007; Brennan et al., 2013b).

What is the mechanism by which *V. fischeri* cells control flagellation? In well-studied species of bacteria, such as *Escherichia coli*, *Salmonella enterica* Typhimurium and *Vibrio cholerae,* flagellar biosynthesis is regulated in a hierarchical, temporal fashion such that the most proximal structural proteins are expressed and assembled before the more distal ones (Chilcott and Hughes, 2000; Prouty et al., 2001). In *V. cholerae,* there are four classes of genes that code for either regulatory or structural proteins (**Figure 3**). The sole Class I gene encodes FlrA, a transcriptional activator that controls expression of Class II genes in a manner that depends upon the alternative sigma factor, σ^54^. Two Class II regulatory proteins, FlrC and the alternative sigma factor σ^28^ (FliA), control expression of Class III and Class IV genes, respectively. Classes II, III, and IV also encode different subunits of the flagellar apparatus. This temporal regulation of gene expression ensures proper, step-wise assembly of the flagellum (**Figure 3**).

Bioinformatic studies suggest that *V. fischeri* cells use regulators similar to *V. cholerae* to control flagellar assembly, and mutagenesis studies thus far have supported this hypothesis (for an extensive list, see Brennan et al., 2013b). Mutations in a few of these genes also cause pleiotropic effects. For example, mutations in the motility regulators *rpoN* (σ^54^) and *flrC* affected bioluminescence, biofilms, and growth in various media (Millikan and Ruby, 2003; Wolfe et al., 2004; Hussa et al., 2007). Additionally, a deletion of the master regulator of flagellar synthesis, *flrA*, affected the expression of a number of genes and proteins unrelated to motility, including a predicted topoisomerase, an ADP-ribosyltransferase similar to the CTX toxin in *V. cholerae* (halovibrin A), phosphoglycerate kinase, a potassium efflux protein, and genes involved in chromosome partitioning (Millikan and Ruby, 2004; Brennan et al., 2013b). These data suggest that motility regulators in *V. fischeri* might be involved in flagellar-independent pathways and predict that these other pathways might also impact colonization. However, the contribution of these motility-independent pathways in host association has yet to be assessed (Millikan and Ruby, 2003; Wolfe et al., 2004; Hussa et al., 2007).

Motility in *V. fischeri* requires the expression of many putative flagellar structural proteins (Brennan et al., 2013b). A few of the flagellin proteins, which polymerize to form the long, external flagellar filament, have been studied in some depth (Millikan and Ruby, 2004). An insertional mutation in *flaA*, which encodes the major flagellin protein (FlaA), resulted in fewer flagella and caused partial defects in motility and colonization (Millikan and Ruby, 2004). The partial defects could be attributed to the presence of at least 5 other flagellin genes in the *V. fischeri* genome. In support of this hypothesis, an insertional mutation in another flagellin gene, *flaD*, also caused a motility defect; conversely, a mutation in the *flaC* flagellin gene had no observable effect on motility (Millikan and Ruby, 2004; Brennan et al., 2013b). To date, no other flagellin genes have been studied in detail. It is not clear whether these “alternative” flagellin proteins are (i) only minor constituents of the flagella, (ii) only utilized in a subpopulation of cells, (iii) specific for the squid association, or (iv) perform yet unknown functions.

Although *V. fischeri* must be motile to colonize the squid, many intriguing questions about this phenotype remain unanswered. For example, many flagellar proteins can be found within light organ exudates, but it is thought that *V. fischeri* is largely aflagellate in the light organ (Ruby and Asato, 1993; Schleicher and Nyholm, 2011). Thus, what is the functional significance, if any, of the presence of these proteins within the light organ? Could these proteins serve as signaling molecules to other *V. fischeri* cells or to the squid? Furthermore, what environmental signals control the loss and/or regeneration of flagella? What are the levels of magnesium associated with different squid tissues, and do the levels impact flagellation in symbiosis? If so, what is the mechanism? If not, are there molecules released by *E. scolopes* or specific environmental cues that dictate the flagellation state of *V. fischeri* cells? Further research into the control of flagellation should shed light on the mechanism by which this important phenotype is altered during the multiple stages of symbiosis.

### CHEMOTAXIS

**FIGURE 4:**
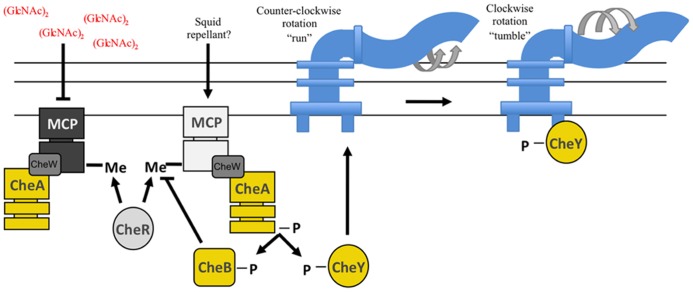
**Predicted chemotaxis pathway in *V. fischeri*.** Methyl-accepting chemotaxis proteins (MCPs) recognize specific molecules found in the environment. A MCP is often physically linked to the sensor kinase, CheA, through the CheW protein. Ligand recognition by the MCP leads to a change in the activity of CheA. Binding of an attractant, such as (GlcNAc)_2_, inhibits CheA kinase activity resulting in a “run.” Conversely, interaction with a repellant promotes CheA autophosphorylation wherein the phosphoryl groups are donated to both CheB and CheY. Phospho-CheY binds to the base of the flagellar motor and causes the flagellum to switch from a counter-clockwise to clockwise rotation. This causes tumbling. The methylesterase, CheB, and the methyltransferase, CheR, both control the methylation state of the MCP allowing a cell to adapt to varying concentrations of chemicals within a chemogradient. Regulators indicated in yellow have been demonstrated to be important for squid colonization (Hussa et al., 2007; Deloney-Marino and Visick, 2012; Mandel et al., 2012).

To identify and reach the colonization-permissive locations within *E. scolopes*, *V. fischeri* cells appear to use chemotaxis, a mechanism bacteria utilize to sense and move toward attractants and away from repellants (reviewed in Manson et al., 1998; Wadhams and Armitage, 2004; Sourjik and Wingreen, 2012). Chemotaxis, a process well-studied in other bacteria, consists of a series of “runs” (smooth swimming) and “tumbles” (for re-orientation). These events depend upon a complex TCS pathway in which receptors, or methyl-accepting chemotaxis proteins (MCPs), are coupled to a SK, CheA, and two downstream RRs, CheY and CheB (Hess et al., 1988; Bourret and Stock, 2002; **Figure 4**). Phospho-CheY directly interacts with the base of the flagellar motor, causing the flagellum to switch its rotation, leading to tumbling and reorientation of the cell (Wadhams and Armitage, 2004). Binding of attractants has the effect of reducing phospho-CheY levels, thereby decreasing tumbling (and increasing smooth swimming). Similarly, deleting *cheY* generates a strain that cannot tumble; therefore, it exhibits a “smooth” run. For a bacterium to continually respond to signals within a chemogradient, it must desensitize and reset the chemotaxis system. To do this, bacteria often control MCP activity by methylating or demethylating specific residues, which activates or deactivates the MCP, respectively (Borkovich et al., 1992; Manson et al., 1998; **Figure 4**). The constitutive methyltransferase, CheR, and the inducible methylesterase, CheB, reversibly control the methylation state of MCPs (Springer and Koshland, 1977; Kehry et al., 1983). Mutation of *cheR* or *cheB* causes cells to exhibit smooth runs or to tumble, respectively, because they cannot adapt to chemogradients (Springer and Koshland, 1977; Stock and Koshland, 1978; Borkovich et al., 1992).

The *V. fischeri* genome contains many genes predicted to be involved in chemotaxis, including the RR, *cheY,* and the methyltransferase, *cheR*. Strains with mutations in *cheY* or *cheR* exhibit “smooth” swimming, similar to *cheY* and *cheR* mutants of *E. coli* (Hussa et al., 2007; Deloney-Marino and Visick, 2012). Importantly, *cheY* and *cheR* mutants fail to compete with wild-type cells for colonization of the squid (Hussa et al., 2007; Deloney-Marino and Visick, 2012). These results suggest that *V. fischeri* cells respond to chemogradients, and that this promotes efficient host colonization (Hussa et al., 2007; Deloney-Marino and Visick, 2012).

Chemotaxis studies performed with *V. fischeri* cells revealed that these bacteria can chemotax to serine, nucleosides, and a variety of sugars, including N-acetylneuraminic acid (NANA) and two chitin components; the monosaccharide, GlcNAc, and the disaccharide, (GlcNAc)_2_ (DeLoney-Marino et al., 2003; Mandel et al., 2012). Interestingly, these three sugars are associated with the squid environment; NANA is found in squid mucous, while GlcNAc and (GlcNAc)_2_ are found within the light organ (Nyholm et al., 2000; Heath-Heckman and McFall-Ngai, 2011). These data suggest that *V. fischeri* cells could use these sugar molecules to chemotax toward the mucous outside the light organ and then into the crypt spaces within the light organ (Nyholm et al., 2000; DeLoney-Marino et al., 2003; Mandel et al., 2012). In support of this, upon exposure to *V. fischeri*, *E. scolopes* expresses a chitin-degrading enzyme in and around the ducts that is predicted to establish a gradient of chitin degradation products, such as (GlcNAc)_2_, that the bacteria can use for chemotaxis (Kremer et al., 2013). Furthermore, prior exposure of *V. fischeri* to (GlcNAc)_2_, such as might occur during symbiotic aggregation, induced a four-fold increase in chemotaxis to this molecule (Kremer et al., 2013). Finally, and perhaps most importantly, Mandel et al. (2012) determined that disruption of the (GlcNAc)_2_ gradient hindered *V. fischeri* from colonizing due to its inability to enter the ducts; the bacteria formed aggregates around the pore, but they rarely entered the light organ.

To guide their migration through the squid, *V. fischeri* cells presumably use MCPs to sense attractants, such as GlcNAc_2_. In other organisms, the number of MCPs can range from 4 in *E. coli* to over 45 in *V. cholerae*, and it is believed that the number of MCPs reflect the complexity of environments a bacterium experiences (Boin and Hase, 2007; Lacal et al., 2010). The *V. fischeri* genome contains 43 putative MCPs, suggesting it has the capability to navigate toward or away from a large repertoire of attractants and repellants, respectively (Ruby et al., 2005; Mandel et al., 2012; Brennan et al., 2013a). Research into these MCPs in *V. fischeri*, however, has proven more difficult than anticipated. Although 19 of the putative MCP genes have been mutated, only one MCP mutant exhibited abnormal chemotaxis toward amino acids *in vitro*, and none exhibited a colonization defect (Mandel et al., 2012; Brennan et al., 2013a). These data suggest that some MCPs may sense the same signal, and/or perhaps MCPs important for *in vitro* and *in vivo* motility have yet to be studied. Identifying the functions of MCPs will surely provide insights into how *V. fischeri* cells direct their movement toward colonization-permissive sites. Furthermore, studying these MCPs may also shed light on host mechanisms and molecules used to promote colonization.

## ON THE DEFENSE: COMBATING ANTIMICROBIALS

Every environment has the potential to be hostile toward a bacterium. This is especially true when a bacterium is exposed to the animal environment, where host immune pathways are implemented to eradicate an unsolicited microbe. As a result, a bacterial symbiont must be able to effectively respond to antimicrobials to promote an interaction with its host. The symbiosis between *E. scolopes* and *V. fischeri* is proving to be a useful model for understanding how beneficial microbes manage antimicrobial challenges received from a host. From initial aggregation outside the light organ to persistent colonization within, *V. fischeri* cells continually interact with antimicrobials and have evolved mechanisms to manage these molecules and thus maintain specificity within the symbiosis (Ruby and McFall-Ngai, 1999; McFall-Ngai et al., 2010; Nyholm and Graf, 2012).

### NITRIC OXIDE

**FIGURE 5:**
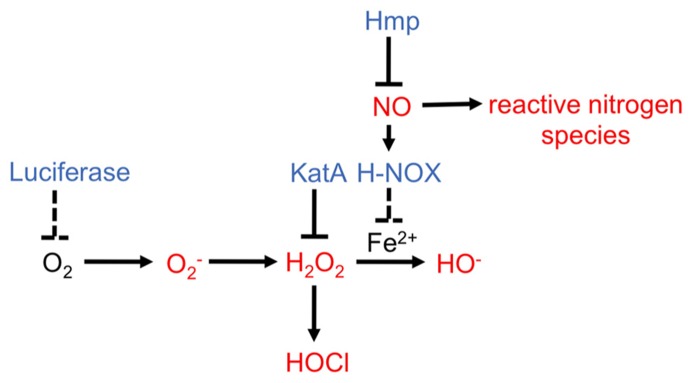
**Reactive oxygen species and reactive nitrogen species pathways and *V. fischeri* proteins potentially involved in modulating synthesis of these antimicrobials.** ROS and RNS are indicated in red (Fang, 2004; Bowman et al., 2011). *V. fischeri* enzymes that have been demonstrated (solid line) or predicted (dashed line) to modulate levels of potential antimicrobial molecules are indicated in blue.

One host molecule experienced by *V. fischeri* during all steps of colonization is nitric oxide (NO), a gaseous, readily diffusible molecule with a wide range of functions (Davidson et al., 2004; Bowman et al., 2011). NO has been studied in mammals, where it controls cellular signaling and, importantly, participates in secondary reactions that produce antimicrobial RNS (Crane et al., 2010; Bowman et al., 2011; **Figure 5**). Presumably, all domains of life produce NO as phylogenetically-distinct organisms contain at least one gene that encodes NO synthase, or NOS (Crane et al., 2010). *E. scolopes*, too, produces an NOS enzyme, as NOS protein and NO molecules were detected within mucous outside the light organ, within the ciliated epithelial fields on the light organ surface, and in the ducts and antechamber of the light organ (Davidson et al., 2004). Surprisingly, after 18 h of colonization by *V. fischeri*, these tissues exhibited a decrease in NOS and NO levels relative to those of aposymbiotic squid (Davidson et al., 2004). This decrease was due to the symbiont’s release of two MAMPs, lipopolysaccharide (LPS) and TCT, a component of the peptidoglycan (Altura et al., 2011). Together, these data suggest that NO plays a key role in the cross-talk between *E. scolopes* and *V. fischeri*.

What is the function of NO within this symbiosis? Within many host environments, NO can participate in reactions that generate antimicrobials; however, *V. fischeri* cells exposed to NO do not exhibit a growth defect, at least not when grown aerobically (Wang et al., 2010a,b). In contrast, squid treated with an NO scavenger allowed *V. fischeri* and even the non-symbiotic relative, *Vibrio parahaemolyticus,* to hyper-aggregate around the light organ (Davidson et al., 2004). Combined, these data suggest that NO might be toxic for *V. fischeri* under particular conditions, but this organism may have developed pathways to sense and resist free NO.

Indeed, *V. fischeri* encodes H-NOX, a protein that, in other bacteria, was predicted to be an NO sensing protein because it binds to NO but, until recently, had few known physiological roles (Boon and Marletta, 2005; Price et al., 2007; Carlson et al., 2010). As expected, *V. fischeri*’s H-NOX did bind NO (Wang et al., 2010a). The novel discovery was that an *hnoX* mutation disrupted *V. fischeri*’s normal transcriptional response to NO exposure, leading to the hypothesis that *hnoX* might sense NO and lead to the detoxification of this molecule during colonization (Wang et al., 2010a). This was not found to be true, as an *hnoX* mutant substantially outcompeted wild-type cells for initiation of colonization, although the difference was diminished after 48 h. Upon inspection of the expression differences in an *hnoX* mutant, it was revealed that, instead of the expected NO defense genes, a set of 10 iron acquisition genes, including hemin receptors, was up-regulated. This suggests that H-NOX usually inhibits iron uptake. In support of this idea, the *hnoX* mutant grew better in hemin-supplemented minimal media than wild-type cells (Wang et al., 2010a).

Why would NO sensing by HNOX lead to a downregulation in the seemingly unrelated iron uptake pathways? The answer to this question remains murky. One possibility is that a high concentration of accumulated intracellular iron in *V. fischeri* within the light organ may be detrimental (Halliwell and Gutteridge, 1984). In fact, it is known that an increase in iron concentrations within a bacterium can lead to the production of harmful hydroxyl radicals through the Fenton reaction, in which H_2_O_2_ is converted to ROS (Halliwell and Gutteridge, 1984; Touati, 2000; **Figure 5**). It was proposed that early NO sensing through H-NOX “primes” *V. fischeri* for the crypt environment, where survival may depend upon the ability of the bacterium to combat the formation of these hydroxyl radicals by controlling the levels of free iron (McCormick et al., 1998; Semsey et al., 2006; Wang et al., 2010a; **Figure 5**). In contrast to this hypothesis, haem uptake genes in *V. fischeri* were upregulated 28 h post inoculation and were required for persistence within the light organ (Septer et al., 2011). Furthermore, a mutation in *glnD*, which led to a growth defect in low iron conditions, caused a defect in squid colonization (Graf and Ruby, 2000). Clearly, iron uptake is a complex process that seems to be partly regulated by NO and H-NOX, although the exact role of these regulators in this pathway remain unclear.

H-NOX responds to NO, but it does not induce expression of enzymes that neutralize NO. How, then, do *V. fischeri* cells defend against antimicrobials produced from NO? The genome of *V. fischeri* encodes additional regulators known to affect the expression of NO detoxification pathways in other bacteria (Rodionov et al., 2005; Spiro, 2007). One of the best characterized regulators is NsrR, a transcriptional repressor that inhibits NO mediators including globins and reductases (Bowman et al., 2011). The most conserved gene within the NsrR regulon is *hmp*, which codes for flavohemoglobin, a NO dioxygenase that eliminates NO by converting it to nitrate (Tucker et al., 2010). Similar to other bacteria, exposure of *V. fischeri* to NO and/or deletion of the repressor, *nsrR*, promoted *hmp* expression (Wang et al., 2010a,b). In addition, an *hmp* mutant exposed to NO exhibited a growth defect and a deficiency in oxygen consumption, consistent with its putative function in NO elimination and NO functioning as an antimicrobial. Furthermore, complementation of *hmp* on a high copy plasmid made the cells hyper-resistant to NO (Wang et al., 2010b). NO detoxification via Hmp was also important for colonization: not only was *hmp* promoter activity induced in response to host-derived NO, but cells deleted for *hmp* exhibited a colonization defect at the aggregation stage. Additionally, treating squid with an NOS inhibitor increased the competitiveness of the *hmp* mutant for colonization (Wang et al., 2010b). Finally, *in vitro* experiments demonstrated that pre-treatment of *V. fischeri* cells with NO reduced the severity of the growth arrest upon a second NO challenge. This result indicates that NO exposure can prime *V. fischeri* for subsequent NO challenges, and suggests that perhaps NO sensed by *V. fischeri* cells at the beginning of colonization may serve as a signal to prepare them for subsequent exposure to NO within the light organ (Wang et al., 2010b).

Although much research has demonstrated the importance of NO in the *V. fischeri/E. scolopes* symbiosis, many intriguing questions remain. For example, what proteins directly sense NO and activate mediators of the NO detoxification response? Are other enzymes besides Hmp involved in detoxifying NO and are they involved in the symbiosis? What exactly is the functional link between NO sensing and iron acquisition in *V. fischeri*? Pursuing these questions is of interest not only to the *E. scolopes*/*V. fischeri* field, but also to the many areas of research that study how host NO production affects colonization by symbiotic and/or pathogenic bacteria (Richardson et al., 2006; Baudouin et al., 2007; Bouchard and Yamasaki, 2008).

### REACTIVE OXYGEN SPECIES

In addition to RNS, the squid produces enzymes predicted to generate ROS (Tomarev et al., 1993; Schleicher and Nyholm, 2011). The light organ and the gills of the squid, tissues known to be exposed to bacteria, produce halide peroxidase (HPO; Tomarev et al., 1993; Weis et al., 1996; Small and McFall-Ngai, 1999; Schleicher and Nyholm, 2011). HPO converts H_2_O_2_ into HOCl, a chemical that is toxic to *V fischeri* and other bacteria (**Figure 5**). Upon colonization by *V. fischeri*, the levels of HPO in host tissues decrease, although the mechanisms behind this remain unknown (Small and McFall-Ngai, 1999). One potential mechanism to manage HOCl levels is through production of a catalase enzyme; this enzyme converts H_2_O_2_ to water and oxygen, thereby lowering the amount of H_2_O_2_ available for conversion to HOCl (Mishra and Imlay, 2012). In *V. fischeri*, mutation of the putative catalase gene, *katA*, caused increased sensitivity to H_2_O_2_ (Visick and Ruby, 1998). Furthermore, the addition of H_2_O_2_ to cells induced *katA* expression, indicating that the bacteria recognize and respond to this antimicrobial. Finally, a *katA* mutant exhibited a defect in competing with the wild-type for colonization, suggesting that reducing H_2_O_2_ levels and/or preventing HOCl formation is important for the symbiosis (Visick and Ruby, 1998).

*E. scolopes* and *V. fischeri* cells produce many other enzymes involved in generating or attenuating ROS, respectively; however, the signals that induce their expression, and the mechanisms by which they might function remain unknown (Stabb et al., 2001; Schleicher and Nyholm, 2011). One unusual pathway hypothesized to reduce ROS is the bioluminescence pathway, due to the requirement for O_2_ by the light-producing enzyme, luciferase. Thus, this pathway could lower levels of oxygen molecules and reduce the potential for their conversion into superoxide ($O_{2}^{-}$; Ruby and McFall-Ngai, 1999; **Figure 5**). Whether bioluminescence, in fact, reduces the levels of ROS within the symbiosis remains to be determined.

### OTHER ANTIMICROBIALS

*E. scolopes* expresses a variety of enzymes that are potentially antimicrobial such as Cathepsin L, chymotrypsin protease, lysozyme, and five peptidoglycan-recognition proteins (PGRPs; Wier et al., 2010; Schleicher and Nyholm, 2011; Collins et al., 2012; Kremer et al., 2013). One PGPR protein, PGPR2, can bind and degrade components of peptidoglycan; however, it seems to play a role in maintenance of the symbiosis rather than as an antimicrobial (Troll et al., 2010). Whereas normally peptidoglycan can serve as an immune stimulant for the host, the PGRP2 enzyme degraded the peptidoglycan components, thereby preventing an inflammatory response, perhaps protecting the host and/or preventing the host from clearing *V. fischeri* cells in the light organ. Whether PGPR2 or any other putative antimicrobial enzyme expressed by the squid is toxic toward *V. fischeri* or other microorganisms, or whether *V. fischeri* has mechanisms to combat these potential antimicrobials remains unknown.

## LIGHT IN A DARK PLACE: BIOLUMINESCENCE

### Lux AND QUORUM SENSING

One of the first characterized and perhaps most striking phenotype exhibited by *V. fischeri* is its ability to bioluminesce, a phenomenon required for a productive symbiosis with the squid (Wei and Young, 1989; Visick et al., 2000). In exchange for nutrients, *V. fischeri* supplies light to *E. scolopes* so that the squid can mask its silhouette cast by moonlight (Jones and Nishiguchi, 2004). This process, known as counterillumination, is hypothesized to protect the squid from predation while it hunts for food at night (Jones and Nishiguchi, 2004). The importance of this phenotype to the symbiosis was established when it was determined that mutants unable to produce light failed to persist in symbiosis (Visick et al., 2000).

**FIGURE 6:**
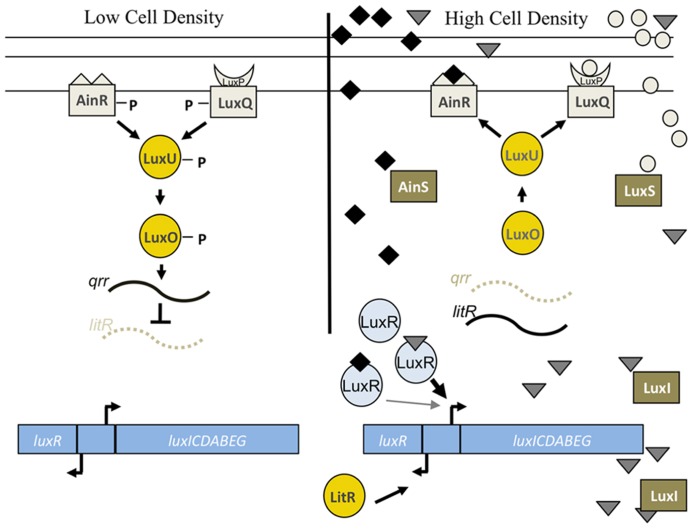
**Lux pathway controlling bioluminescence in *V. fischeri.*** At low cell density, the sensor kinases AinR and LuxP/Q are predicted to exhibit net kinase activity leading to the phosphorylation of LuxU and subsequent phosphotransfer to LuxO. Phospho-LuxO induces the expression of the inhibitory sRNA, *qrr1*, which leads to the degradation of *litR* mRNA. LitR is the transcriptional activator of *luxR*, which encodes a protein required for expression of the *luxCDEBAG* operon. Thus, at low cell density, *litR* translation is inhibited and the cells do not produce high levels of light. At high cell density, two distinct autoinducer molecules made by AinS (C8-HSL, diamonds) and LuxS (AI-2, circles) are predicted to be at sufficient concentrations to switch the activity of the SKs from net kinase to net phosphatase activity. This leads to dephosphorylation of the downstream regulators, *litR* translation, and transcription of *luxR*. LuxR, in conjunction with the autoinducer produced by LuxI (3-oxo-C6-HSL, triangles), leads to the transcription of the *lux* operon and bioluminescence (reviewed in Stabb et al., 2008). LuxR is also predicted to weakly bind to C8-HSL, which allows for the initiation of *luxCDABEG* expression. See text for caveats to this model.

The structural proteins necessary for light generation (LuxCDABEG) are encoded by the *lux* operon (Gray and Greenberg, 1992; **Figure 6**). Numerous other proteins control light production, including the first gene in the *lux* operon, *luxI*, which encodes an autoinducer synthase, and the divergently transcribed gene, *luxR*, which encodes a transcription factor that regulates the *lux* operon. LuxI synthesizes a pheromone, 3-oxo-C6-HSL, that promotes *lux* transcription by binding to and activating LuxR (**Figure 6**; for reviews, see Stabb et al., 2008; Ng and Bassler, 2009). As a result, these regulators participate in a positive feedback loop, such that the LuxR-3-oxo-C6-HSL complex promotes synthesis not only of the Lux enzymes, but also of more 3-oxo-C6-HSL, thus amplifying light production.

LuxR itself is controlled at the level of transcription via input from a complex phosphorelay pathway (see reviews Stabb et al., 2008; Ng and Bassler, 2009; **Figure 6**). This pathway is comprised of two SKs, AinR and LuxP/Q, and additional downstream regulators, including the histidine phosphotransferase, LuxU, and the σ^54^-dependent RR, LuxO. At low cell density, the SKs function as kinases to autophosphorylate and serve as phosphodonors to a single phosphotransferase, LuxU, which, when phosphorylated, can donate a phosphoryl group to LuxO. At high cell density, the phospho-transfer pathway is reversed, with the SKs functioning as phosphatases to remove the phosphoryl group from LuxU (and LuxO). When LuxO is phosphorylated (at low cell density), it activates expression of the sRNA, *qrr1,* which inhibits the translation of the *litR* mRNA (Miyashiro et al., 2010). LitR is the direct transcriptional activator of *luxR*; thus, inhibition of *litR* leads to an inhibition of bioluminescence (Fidopiastis et al., 2002; Miyashiro et al., 2010). When LuxO is dephosphorylated (at high cell density), *qrr1* levels decrease, LitR is translated, *luxR* is transcribed, and the *lux* operon is expressed.

The SKs in the phosphorelay, AinR and LuxPQ, are predicted to sense and respond to the presence of specific pheromones, C8-HSL and AI-2, produced by the autoinducer synthases AinS and LuxS, respectively (reviewed in Stabb et al., 2008). It is predicted that these pheromones accumulate at high cell density, causing the SKs to function as phosphatases and thus promote light production. However, the two inputs do not equally control bioluminescence. For example, an *ainS* mutation caused a severe bioluminescence defect both *in vitro* and in the squid, while a *luxS* mutation did not dramatically alter any bioluminescence phenotype (Lupp et al., 2003; Lupp and Ruby, 2004, 2005). Similarly, colonization experiments found that the *ainS* mutant exhibited defects in both initiation and persistence, while the *luxS* mutant did not (Lupp and Ruby, 2004, 2005). This suggests that the AinS/R branch is more important for controlling light production and colonization than the LuxS-LuxP/Q branch. One possible explanation for this differential importance is suggested by the finding that the AinS-produced C8-HSL can interact directly with LuxR, albeit at a lower affinity than occurs with 3-oxo-C6-HSL, produced by LuxI (Schaefer et al., 1996; Egland and Greenberg, 2000); thus, C8-HSL can exerts its impact both directly and indirectly to control *lux* expression and therefore light levels (Lupp et al., 2003). These and other data support a model in which the C8-HSL-LuxR complex initiates transcription of the *lux* operon, while 3-oxo-C6-HSL-LuxR ultimately takes over as the critical player that promotes positive feedback of *lux* transcription.

Although the above model fits with what is known about the Lux pathway in other bacteria, the roles of the upstream players in *V. fischeri* remain poorly understood. For example, a mutation in the C8-HSL synthase gene, *ainS,* severely impacted bioluminescence, yet a deletion in *ainR,* the SK predicted to respond to C8-HSL, appeared to exert only a minor effect on luminescence (Lupp et al., 2003; Lupp and Ruby, 2004; Ray and Visick, 2012). These results may indicate that the role of C8-HSL in activating LuxR may be more important than its role in controlling the AinR-mediated phosphorelay.

Surprisingly, the bioluminescence phenotypes of some regulators do not correlate with the predicted colonization phenotypes. For example, a *litR,* mutant exhibited a bioluminescence defect but was not impaired for colonization; in fact, this mutant colonized *E. scolopes* better than wild-type in competition experiments after 48 h (Fidopiastis et al., 2002; Miyashiro et al., 2010). However, in a different study, the same *litR* mutant exhibited a colonization disadvantage at an earlier, 12 h time point (Lupp and Ruby, 2005). Additionally, although mutants deleted for the negative regulators of bioluminescence, *luxO* or *qrr1,* exhibited increased bioluminescence *in vitro,* as expected, they exhibited a defect in colonization when competed with wild-type cells (Hussa et al., 2007; Miyashiro et al., 2010). These results suggest that bioluminescence regulators may have bioluminescence-independent functions. In support of this hypothesis, it was found that LuxO and LitR have complex regulons: LuxO controls many genes outside of the canonical Lux pathway, and LitR contributes to controlling whether the cells secrete or import acetate for metabolism, known as the acetate switch (Fidopiastis et al., 2002; Lupp and Ruby, 2005; Hussa et al., 2007; Studer et al., 2008).

Lastly, an interesting observation made about light production in *V. fischeri* is that most environmental strains of this bacterium are visibly bioluminescent when grown in culture, but strains isolated from *E. scolopes* are “dim,” meaning they are bioluminescent but do not emit visible light outside of the squid (Boettcher and Ruby, 1990; Stabb et al., 2008). Furthermore, an experimental evolution model has revealed a correlation between increased colonization and decreased light production (Schuster et al., 2010). These findings indicate that some aspect of the light organ environment enriches for dim strains of *V. fischeri*. Perhaps this is not surprising, as producing bioluminescence is energetically taxing (Bourgois et al., 2001). The luciferase enzyme, consisting of the LuxA and LuxB heterodimer, can constitute up to 5% of total protein in visibly luminescent cells (Hastings et al., 1965). Furthermore, under particular growth conditions, expression of the *lux* operon causes a growth defect (Bose et al., 2008). Therefore, light production must in some way benefit *V. fischeri* both inside and outside a host. Numerous possibilities have been proposed, including the removal of oxygen from the environment, which could decrease the production of ROS (e.g., see Stabb et al., 2008). Additionally, because the squid can detect the bacterial bioluminescence, it is hypothesized that the squid may play an active part in maintaining a population of *V. fischeri* cells with a particular bioluminescence phenotype (Tong et al., 2009; Heath-Heckman et al., 2013). However, no single explanation has yet been established for how strains with particular luminescence levels are enriched within the squid, or how brightly luminescent bacteria survive in other marine environments.

### ADDITIONAL REGULATORS OF BIOLUMINESCENCE

It has long been known that squid symbionts produce a level of light during symbiosis that is about ~1000X brighter than that produced in culture (Boettcher and Ruby, 1990). These data suggest that there are squid specific-signals that affect luciferase production, and that *V. fischeri* might sense these signals using regulators that are outside of the canonical Lux pathway. In fact, a variety of environmental conditions affect the ability of *V. fischeri* to produce light, all of which might have the potential to be sensed by non-Lux regulators. These putative signals that affect bioluminescence include changes in oxygen levels, osmolarity, Mg^2^^+^ levels, and iron levels (Stabb et al., 2004; Bose et al., 2007; Lyell et al., 2010; Lyell and Stabb, 2013; Septer et al., 2013).

One key non-Lux regulator is ArcA, a RR predicted to function as a transcription factor when phosphorylated by its cognate SK, ArcB (Iuchi et al., 1990; Iuchi and Lin, 1992; Georgellis et al., 1997, 2001; Pena-Sandoval et al., 2005). In *V. fischeri,* a deletion of *arcA* caused a dramatic ~500 fold increase in bioluminescence in culture, resulting in light levels that were similar to the levels found during symbiosis (Bose et al., 2007). ArcA appears to exert a direct effect on *lux* transcription by binding to a site upstream of the *lux* operon (Bose et al., 2007). These results suggest that ArcA functions as a transcriptional inhibitor of luminescence genes under culture conditions, and that this inhibition is relieved once *V. fischeri* is inside the light organ. The effect of the *arcA* mutation on bioluminescence, however, was dependent on the presence of an intact *luxI* gene (Septer and Stabb, 2012). This result indicates ArcA primarily functions to inhibit the positive feedback loop that relies on the LuxI-synthesized molecule, 3-oxo-C6-HSL (Septer and Stabb, 2012).

In other systems, the ArcA/B pathway is predicted to sense and respond to the redox state of the cell (reviewed in Malpica et al., 2006). In *E. coli*, reducing conditions sensed by ArcB lead to the phosphorylation and thus activation of ArcA, while oxidizing conditions generate unphosphorylated ArcA. Thus, it has been proposed that this two-component pathway in *V. fischeri* senses the oxidized state in the light organ, leading to unphosphorylated ArcA and a de-repression of bioluminescence (Bose et al., 2007); however, experimental evidence of this has yet to be found. Furthermore, although an *arcA* mutation amplified bioluminescence levels, ArcA might not be the only non-Lux regulator of bioluminescence. For example, a recent transposon screen identified mutations in additional genes that led to an increase in light production (Lyell et al., 2010; Lyell and Stabb, 2013). Whether these genes are directly involved in controlling light production, and/or whether they sense particular environments to regulate bioluminescence remain an active area of research.

## CONCLUSION

For such a seemingly simple symbiosis, the interaction between *V. fischeri* and *E. scolopes* requires numerous, complicated regulatory pathways to promote host specificity and colonization. It should be noted that this review focuses only on regulators within *V. fischeri* known to be important for colonization, yet a considerable repertoire of information exists that details host specific pathways that are integral for the symbiosis to occur (see reviews Nyholm and McFall-Ngai, 2004; McFall-Ngai et al., 2012; Stabb and Visick, 2013). Although much information exists about the *V. fischeri* pathways described in the above sections, including biofilms, chemotaxis, responses to antimicrobials, and light production, many discoveries within these signaling cascades have uncovered yet more questions that can still be addressed. For example, what molecular pathways allow *V. fischeri* to outcompete other non-symbiont bacteria during aggregate formation? How is flagellation and deflagellation controlled during the various steps of colonization? What regulators sense antimicrobials and lead to their detoxification? And lastly, what environmental signal within the light organ promotes bioluminescence? All of these subjects are active areas of research, and they will surely uncover new mechanisms that will expand the knowledge of how bacteria and a host establish a life-long, beneficial relationship.
